# Identification of Youthful Neurocognitive Trajectories in Adults Aging with HIV: A Latent Growth Mixture Model

**DOI:** 10.1007/s10461-021-03546-9

**Published:** 2021-12-08

**Authors:** Rowan Saloner, Judith D. Lobo, Emily W. Paolillo, Laura M. Campbell, Scott L. Letendre, Mariana Cherner, Igor Grant, Robert K. Heaton, Ronald J. Ellis, Scott C. Roesch, David J. Moore, CHARTER Study Group

**Affiliations:** 1 San Diego Joint Doctoral Program in Clinical Psychology, San Diego State University/University of California, San Diego, CA, USA; 2 Department of Psychiatry, HIV Neurobehavioral Research Program, University of California, San Diego, 220 Dickinson Street, Suite B, Mail Code 8231, San Diego, CA 92103-8231, USA; 3 Department of Neurology, University of California, San Francisco, Memory and Aging Center, San Francisco, CA, USA; 4 Department of Neurosciences, University of California, San Diego, San Diego, CA, USA; 5 Department of Psychology, San Diego State University, San Diego, CA, USA

**Keywords:** SuperAging, HIV-associated neurocognitive disorder, Growth mixture model, Cognitive reserve, Comorbidity burden

## Abstract

Despite the neurocognitive risks of aging with HIV, initial cross-sectional data suggest a subpopulation of older people with HIV (PWH) possess youthful neurocognition (NC) characteristic of SuperAgers (SA). Here we characterize longitudinal NC trajectories of older PWH and their convergent validity with baseline SA status, per established SuperAging criteria in PWH, and baseline biopsychosocial factors. Growth mixture modeling (GMM) identified longitudinal NC classes in 184 older (age ≥ 50-years) PWH with 1–5 years of follow-up. Classes were defined using ‘peak-age’ global *T*-scores, which compare performance to a normative sample of 25-year-olds. 3-classes were identified: Class 1_*Stable Elite*_ (*n* = 31 [16.8%], high baseline peak-age *T*-scores with flat trajectory); Class 2_*Quadratic Average*_ (*n* = 100 [54.3%], intermediate baseline peak-age *T*-scores with u-shaped trajectory); Class 3_*Quadratic Low*_ (*n* = 53 [28.8%], low baseline peak-age *T*-scores with u-shaped trajectory). Baseline predictors of Class 1_*Stable Elite*_ included SA status, younger age, higher cognitive and physiologic reserve, and fewer subjective cognitive difficulties. This GMM analysis supports the construct validity of SuperAging in older PWH through identification of a subgroup with longitudinally-stable, youthful neurocognition and robust biopsychosocial health.

## Resumen

A pesar de los riesgos neurocognitivos de envejecer con VIH, datos transversales iniciales sugieren que una subpoblación de personas con VIH (PCV) de edad mayor posee neurocognición (NC) juvenil, característica de los Súper-Ancianos (SA). Aquí nosotros caracterizamos trayectorias longitudinales de NC en PCV mayores y su validez convergente con su status de referencia de SA, según los criterios establecidos en PCV, y factores biopsicosociales en la base de referencia. El modelo de mezclas Gaussianas (GMM) identificó clases longitudinales de NC en 184 PCV mayores (edad ≥ 50-años) con 1–5 años de seguimiento. Las clases fueron definidas utilizando puntuaciones-T (*T*-scores) globales de “edad pico”, que comparan el desempeño con una muestra normativa de personas de 25 años de edad. 3-clases fueron identificadas: Clase 1_*Élite Estable*_ (*n* = 31 [16.8%], puntuaciones-T de edad pico de referencia altas con trayectoria plana; Clase 2_*Promedio Cuadrático*_ (*n* = 100 [54.3%], puntuaciones-T de edad pico de referencia intermedias con trayectoria en forma de u); Clase 3_*Cuadrática Baja*_ (*n* = 53 [28.8%], %], puntuaciones-T de edad pico de referencia bajas con trayectoria en forma de u). Los predictores de referencia de la Clase 1_*Élite Estable*_ incluyen estatus de SA, edad mas joven, reserva cognitiva y fisiológica superior, y menos dificultades cognitivas subjetivas. Este análisis GMM apoya la validez del constructo de Súper-Envejecimiento en PCV mayores mediante la identificación de un subgrupo longitudinalmente estable, neurocognición juvenil y una robusta salud biopsicosocial.

## Introduction

In the U.S. and other developed countries, HIV is no longer considered a rapidly debilitating terminal illness, but rather a chronic medical condition when treated with modern antiretroviral therapy (ART) [1]. Despite increased longevity in the ART-era, geriatric syndromes such as neurocognitive impairment (NCI) and frailty manifest at younger ages and may accumulate at faster rates in persons with HIV (PWH) compared to HIV-seronegative adults [2, 3]. NeuroHIV investigators have accordingly developed models of premature and accelerated aging to characterize the excess risk for age-related central nervous system (CNS) complications in older PWH [4–7]. Aging with HIV appears to exert a heightened neurotoxic effect, as evidenced by accelerated rates of cortical and subcortical brain atrophy [8–10] and excessive levels of neuroinflammation [11, 12]. The source of these neurological vulnerabilities is likely multifactorial, as older PWH have higher rates of biological and psychosocial risk factors for neurobehavioral decline [13, 14].

Age is commonly identified as a risk factor for HIV-associated NCI at the cross-sectional level, however one to two-thirds of older PWH do not meet criteria for NCI and synergistic effects of age and HIV on NCI are not consistently detected [15–17]. Although a substantial proportion of older PWH do not exhibit overt neurocognitive deficits, neuroHIV studies generally do not consider a full range of inter-individual differences in neurocognition (e.g., low average to superior) within this neurocognitively unimpaired group. Aging is associated with increased heterogeneity across most health-related outcomes [18, 19] and the range of unimpaired neurocognitive performance is widened with age-based neuropsychological test score corrections. Differentiating patterns of neurocognition within unimpaired individuals, such as separating out typical neurocognitive aging from superior neurocognitive aging, can enhance understanding of the factors that promote sustained neurocognition compared to factors that ward off NCI but do not prevent “normal” age-related decline.

Neurocognitive aging studies in elders without HIV have recognized the heterogeneous nature of neurocognitive aging and some data suggest that several subpopulations may exist within the broader group of older adults with unimpaired neurocognition. Specifically, there is growing evidence that a subgroup of elders without HIV possess youthful neurocognitive abilities [19]. These individuals, termed cognitive *SuperAgers* (SA), may be resilient to expected age-related neurocognitive decline and they display more robust neurological and psychological functioning compared to the subgroup of cognitively average, but non-super, peers [20]. Despite the neurocognitive hazards of aging with HIV, prior work has also identified a subgroup of older (≥ 50-years) PWH who exhibit comparable cross-sectional neurocognitive performance to that of a healthy 25-year-old (estimated ~ 17% of older PWH) [21]. Compared to their cognitively average and cognitively impaired counterparts with HIV, these SA with HIV exhibit better functioning on key biopsychosocial indicators, including less comorbidity burden and self-reported cognitive and depressive symptoms, as well as higher levels of cognitive reserve.

The initial investigation of SA in PWH highlights the ecological relevance of differentiating SA from cognitively average older PWH, yet these cross-sectional data do not address whether SA with HIV maintain stable neurocognition over time. Characterizing longitudinal trajectories of neurocognitive functioning is essential for unmasking a subgroup of older PWH with superior/peak neurocognitive abilities that are stable across time. Thus, the present study applied latent growth mixture modeling (GMM) to characterize trajectories of youthful neurocognitive aging in a multi-site, national cohort of older PWH. GMM is a person-centered approach that facilitates the identification of latent longitudinal classes, which account for unobserved intercept and slope heterogeneity in the entire sample, and predictors can be specified in GMM to explain inter-class differences in neurocognitive change [22, 23]. Within this GMM framework, we examined the degree to which baseline SuperAging classifications and baseline indicators of biopsychosocial health at baseline (e.g., cognitive reserve, physiologic reserve, depression) converged with longitudinal classes of neurocognitive aging in PWH. We hypothesized that longitudinal neurocognitive patterns would be heterogeneous and latent classes would reflect theoretical trajectories of neurocognitive aging, including a neurocognitively elite and stable subgroup. We also hypothesized that individuals classified as SA at baseline would have the highest odds of membership in longitudinal class(es) defined by elite and stable neurocognition. Similarly, we expected individuals with better baseline biopsychosocial functioning to have higher odds of membership in better longitudinal class(es).

## Methods

### Participants

Participants included 184 older PWH enrolled in the CNS HIV Antiretroviral Therapy Effects Research (CHARTER) study [24] from 2003 to 2017. As a multi-site study, CHARTER participants were drawn from six participating university centers: Johns Hopkins University (Baltimore, MD, *n* = 34); Mt. Sinai School of Medicine (New York, NY, *n* = 42); University of California at San Diego (San Diego, CA, *n* = 18); University of Texas Medical Branch (Galveston, TX, *n* = 40); University of Washington (Seattle, WA, *n* = 25); and Washington University (St. Louis, MO, *n* = 25). To be included in the present longitudinal analysis, participants must have been aged 50 years or older at their baseline visit and must have completed at least 2 additional CHARTER study visits, which occurred in 6-month follow-up intervals (1200 total visits; median = 6 visits; range = 3–10 visits). Of the 1200 observations, 1103 (92%) reflect novel observations that were not included in our prior analysis of SA in HIV [19]. All participants completed a blood draw and comprehensive neuromedical, neurobehavioral, and neuropsychiatric examinations at each study visit. All CHARTER study procedures were approved by local Institutional Review Boards (IRBs) and all participants provided written informed consent. CHARTER participants with baseline conditions that “confounded” the interpretation of neuropsychological test data and its association with HIV disease were excluded from analysis [24, 25]. Confounding conditions included history of severe learning disability, diagnosis of a psychotic (e.g., schizophrenia) or mood disorder with psychotic features, and major neurological conditions (e.g., epilepsy). Visits were also excluded if participants had a positive urine toxicology screen for substance use (except marijuana) or Breathalyzer test for alcohol on the day of testing.

### Neuropsychological Evaluation

At each timepoint, participants completed a standardized battery of neurocognitive tests in domains most affected in HIV: verbal fluency, executive functioning, processing speed, learning, delayed recall, attention/working memory, and motor skills [24, 26]. Individual raw test scores were first corrected for practice effects associated with repeat testing [27]. Scores were then converted to demographically-adjusted *T*-scores (mean = 50, standard deviation = 10) in which the age of 25, when most fluid neurocognitive capacities peak [28–30], was substituted for chronological age in demographic correction formulas [21]. These “peak-age” *T*-scores consequently compare an individual’s neurocognitive performance to normative standards for 25-year-olds of the same education, sex, and race/ethnicity. For example, an individual with a peak-age *T*-score of 40 would have performance 1 standard deviation below the mean of 25-year-olds matched on education, sex, and race/ethnicity. Global peak-age *T*-scores, which reflect the average peak-age *T*-score across the entire test battery, were used as indicator variables in GMM. Global peak-age *T*-scores were selected to estimate longitudinal neurocognitive change (as opposed to traditional chronological age-corrected global *T*-scores and demographically-uncorrected global scaled scores) because: (1) chronological age-based adjustments can mask the influence of age on neurocognitive change over time; (2) peak-age *T*-scores have the added benefit of comparing an individual’s level of performance to youthful expectations while still adjusting for other salient demographic factors.

In addition to estimating longitudinal neurocognitive change, we employed our criteria for SuperAging in PWH [21] to classify participants into three neurocognitive groups at baseline: SA, cognitively normal (CN) for their actual age, or cognitively impaired (CI). Participants were classified as SA at baseline if their peak-age global performance was within normative expectations (i.e., ≥ −1SD from the mean of the 25-year-old normative sample) and they did not exhibit any isolated impairments for individual domains based on traditional, chronological age-based norms. Individuals who were not classified as SA at baseline were either classified as CN or CI based on the traditional, chronological age-based deficit score approach for classifying global neurocognitive impairment in HIV [26, 31].

### Cognitive Reserve and Self-reported Cognitive Symptoms

Cognitive reserve was measured using standardized scores from the Reading subtest of the Wide Range Achievement Test, version 4 (WRAT4) [32], a validated estimate of premorbid verbal intelligence robust to neurocognitive decline and a standard proxy for cognitive reserve in older HIV-seronegative adults and PWH [33–35]. Self-reported cognitive symptoms were assessed with the Patient’s Assessment of Own Functioning Inventory (PAOFI), a 33-item self-report measure used to measure perceived cognitive symptoms in everyday life [36]. Items endorsed as fairly often or greater are considered clinically-significant cognitive symptoms. The continuous PAOFI total score is the number of self-reported, clinically-significant cognitive symptoms in everyday life.

### Physiologic Reserve via Neuromedical Evaluation

The comprehensive neuromedical examination assessed for clinical deficits relevant to HIV and geriatric health. Based on established methods for constructing a frailty index [37–39], a cumulative physiologic reserve variable was composed of 39 unique health variables encompassing a range of physiologic systems, including routine clinical laboratory measures (e.g., glucose, lipids), medical comorbidities (e.g., hepatitis C co-infection, diabetes), and indicators of HIV-disease severity. Each health variable was dichotomized as normal or deficient (normal = “1”; deficit = “0”) based on criteria from previous HIV studies [40, 41] (see Table 1) and physiologic reserve index scores were constructed by dividing the total sum of normal health variables by the total number of available variables, with a possible range of 0 (all 39 deficits) to 1 (no deficits). Thus, higher scores are reflective of higher levels of physiologic reserve.

### Psychiatric Evaluation

The structured Composite International Diagnostic Interview (CIDI) [42] was administered to ascertain DSM-IV diagnoses of current and lifetime mood and substance use disorders. Mood symptoms in the past two weeks were assessed with the Beck Depression Inventory-II (BDI-II) [43].

### Statistical Analysis

As an extension of latent growth curve modeling and latent class analysis, GMM identifies subgroups of individuals that share a common longitudinal pattern. GMM can estimate continuous linear and non-linear latent trajectories of change while simultaneously inferring categorical subgroup (i.e., latent class) membership based on unobserved heterogeneity in trajectories [22, 23]. The present study employed GMM to model latent growth classes of global peak-age *T*-scores measured at the 10 study timepoints, occurring in 6-month intervals. Longitudinal neurocognitive patterns were defined by a latent intercept, representing global peak-age *T*-scores at baseline, and latent slope parameters reflecting an underlying neurocognitive growth process.

GMM analyses were conducted in *Mplus* Version 8.6 [44]. To first determine the best base model for change, unconditional (without covariate specification) latent growth models tested an intercept only, linear, quadratic, cubic, or latent basis model. After identification of the best base model, we iteratively compared 1- to 4-class unconditional GMMs to determine the optimal number of latent classes. For each solution, the best log-likelihood was replicated in order to avoid convergence at a local maximum. The best-fitting solution was determined based on a combination of: (1) statistical fit indices, specifically Akaike information criterion (AIC), sample size-adjusted Bayesian information Criterion (ssBIC), Lo-Mendell-Rubin likelihood ratio test (LMRT), and entropy; (2) adequate class size, with recommendations of at least 25 individuals per class and each class representing at least 5% of the total sample [45]; (3) theoretical interpretability of classes; and 4) model parsimony.

After the optimal number of classes was identified, classes were substantively interpreted based on examination of latent intercept and slope parameters. Wald *χ*^2^-tests examined the concordance of baseline neurocognitive classifications (i.e., SA, CN, and CI) with latent longitudinal class membership in order to examine the convergent validity of SA criteria with longitudinal neurocognitive patterns and further characterize latent class interpretation. Last, a “3-step” approach modeled class membership as a function of baseline demographic and clinical covariates in multinomial logistic regression. Class membership was assigned prior to inclusion of covariates in order to prevent covariates from altering the structure of latent classes and influencing final class membership [46, 47]. Factors that were univariably associated with latent classification at *p* < 0.10 were included as covariates. Covariates that failed to discriminate latent classification at *p* < 0.10 in the multinomial logistic analysis were removed and models were re-estimated. Missing data patterns were analyzed and variables significantly associated with missing data were also included as auxiliary variables [48]. For all models, full-information maximum likelihood estimation was used to account for missing data [44, 49].

## Results

### Participant Characteristics

The full sample of 184 PWH was 82% male with a mean baseline age of 52.9 years (age range: 50–68) and mean education of 13.1 years. With regard to race/ethnicity, the overall sample was 46% non-Hispanic Black, 43% non-Hispanic White, 9% Hispanic, and 2% other. Baseline neurocognitive status classification rates were comparable to our larger cross-sectional study of SuperAging, with 19% of participants classified as SA (*n* = 35), 47% classified as CN (*n* = 87), and 34% classified as CI (*n* = 62). With regard to ART use, 82% of participants were actively on ART medication, 11% reported past use of ART, and 7% were ART-naïve. Current CD4 counts (median = 475 cells/mm^3^) were on average substantially higher than nadir CD4 counts (median = 108.5 cells/mm^3^). The 57% rate of viral suppression (i.e., undetectable plasma HIV RNA) at baseline was comparable to the overall CHARTER cohort and reflective of the broad period of data collection (2003 to 2017). Additional descriptive statistics for the full sample are presented in Table 2.

### Missing Data

For the 9 follow-up visits, the percent of participants with available neucognitive data ranged from 39 to 77%. The lowest covariance coverage for each pair of indicator variables was 0.315, which was above the minimum threshold of 0.10 for model convergence and indicative of acceptable levels of missing data. To determine missing data patterns, the total number of missing timepoints per participant was calculated and associations between number of missing timepoints and study variables were conducted. A higher number of missing timepoints was associated with less education (spearman’s rho = − 0.17, *t* = − 2.14, *p* = 0.034) and a later baseline study date (spearman’s rho = 0.32, *t* = 5.89, *p* < 0.001). Years of education and baseline study date were accordingly specified as auxiliary covariates in GMM with full information maximum likelihood to help reduce potential parameter estimate biases caused by missing data.

### Optimal Latent Trajectory Class Solution

Goodness-of-fit indices supported a quadratic growth model with intercept and slope parameter variances constrained equal across classes as the best base model for change. Table 3 presents the AIC, ssBIC, LMRT, entropy, and class sizes for 1- to 4-class solutions for the quadratic growth model. The AIC and ssBIC metrics were near equivalent within each solution and progressively decreased with higher class solutions, although the magnitude of these changes became smaller with higher class solution comparisons. All models exhibited strong class separation based on entropy. Entropy was lower in the 2-class solution compared to the 3- and 4-class solutions, whereas entropy values were comparable between the 3- and 4-class solutions. The LMRT value was significant for the 2-class solution compared to the 1-class solution, suggesting improved model fit based on log-likelihood in the 2-class solution, but was not significant for the 3- (vs. 2) and 4-class (vs. 3) solutions. Although the two smallest-sized classes in the 4-class solution were each greater than 5% of the total sample, the class sizes were smaller than the recommended minimum class size of 25 and were underpowered for conditional analyses. Given the class size limitations of the 4-class solution and the AIC, ssBIC, and entropy indicators favoring the 3-class solution over the 2-class solution, the 3-class solution was selected as the best fitting model.

**Fig. 1:**
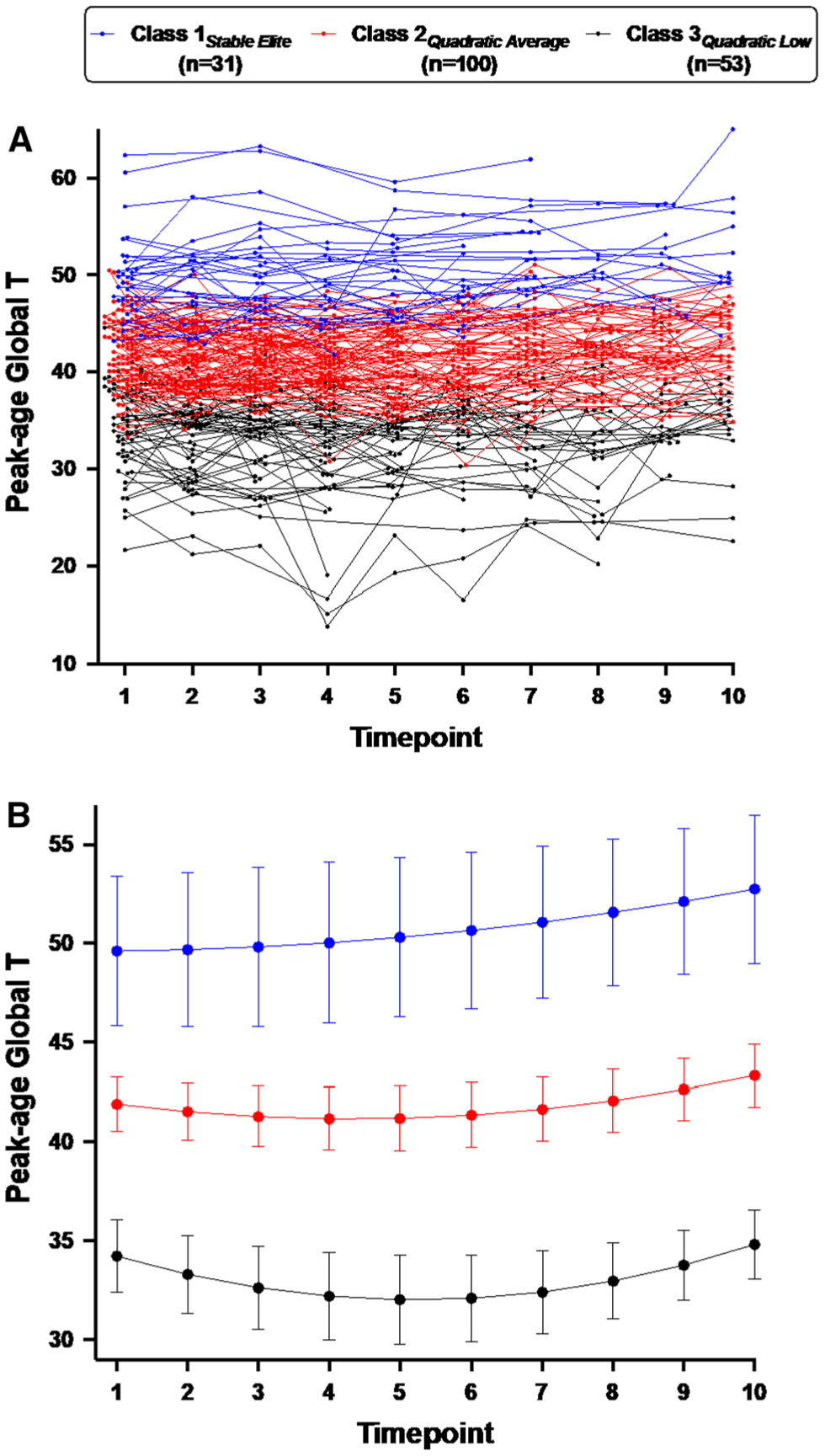
Trajectory plots are coded by the three latent trajectory classes: Class 1_*Stable Elite*_ (blue), Class 2_*Quadratic Average*_ (red), Class 3_*Quadratic Low*_ (black). **A** Spaghetti plot of individual peak-age global *T*-scores (*y*-axis) across the 10 study timepoints (*x*-axis), which occurred in 6-month intervals. **B** Estimated trajectory means by class membership derived from growth mixture modeling. Error bars represent 95% confidence intervals

Figure 1A presents a spaghetti plot of individual longitudinal neurocognitive patterns and Fig. 1B presents the estimated timepoint means by class membership. The three longitudinal classes identified for global peak-age *T*-score were: (1) Class 1_*Stable Elite*_ (16.8% of the sample), whose members exhibited stable, high levels of global peak-age *T*-scores (intercept [SE] = 49.62 [0.61], *p* < 0.001; linear [SE] = 0.04 [0.26], *p* = 0.894; quadratic [SE] = 0.04 [0.04], *p* = 0.361) that generally fell within the range of normative expectations for 25-year-olds (i.e., peak-age *T* ≥ 40); (2) Class 2_*Quadratic Average*_ (54.3% of the sample), whose members on average exhibited a quadratic, shallow “u-shaped” trajectory (intercept [SE] = 41.91 [0.70], *p* < 0.001; linear [SE] = − 0.45 [0.17], *p* = 0.007; quadratic [SE] = 0.07 [0.02], *p* < 0.001) with most individual trajectories fluctuating within the 30 to 50 range of peak-age *T*-scores; and (3) Class 3_*Quadratic Low*_ (28.8%), whose members also on average exhibited a quadratic “u-shaped” trajectory (intercept [SE] = 34.22 [0.93], *p* < 0.001; linear [SE] = − 1.04 [0.29], *p* < 0.001; quadratic [SE] = 0.12 [0.03], *p* < 0.001) with most individual trajectories fluctuating within the 20 to 40 range of peak-age *T*-scores.

### SA Criteria and Latent Group Membership

Baseline neurocognitive classifications (i.e., SA, CN, and CI) were validated against the latent longitudinal classes with Wald *χ*^2^-tests. Overall, baseline neurocognitive status significantly segregated by longitudinal class (*χ*^2^ = 100.26, *p* < 0.0001). Each baseline status comprised at least 63% of a respective longitudinal class. Class 1_*Stable Elite*_ was composed of 65% SA (*n* = 20) and 35% CN (*n* = 11); Class 2_*Quadratic Average*_ was composed of 63% CN (*n* = 63), 22% CI (*n* = 22), and 15% SA (*n* = 15); and Class 3_*Quadratic Low*_ was composed of 75% CI (*n* = 40) and 25% CN (*n* = 13). Since global performance at the baseline timepoint was used in neurocognitive status classifications and as the intercept indicator in the GMM, a secondary GMM model removed baseline performance as an indicator variable. This allowed us to examine the relationship of baseline neurocognitive status to longitudinal classes that were defined solely on data from follow-up timepoints. The 3-class solution based only on follow-up data exhibited comparable class separation (entropy = 0.891) and class sizes (Class 1_*Stable Elite*_ = 27 [15%], Class 2_*Quadratic Average*_ = 102 [55%], Class 3_*Quadratic Low*_ = 55 [30%]) to the 3-class solution that included baseline performance. Only 8 total participants shifted class membership, with 4 (3 SA, 1 CN) shifting from Class 1_*Stable Elite*_ to Class 2_*Quadratic Average*_, 3 (2 CN, 1 CI) shifting from Class 2_*Quadratic Average*_ to Class 3_*Quadratic Low*_, and 1 (1 CI) shifting from Class 3_*Quadratic Low*_ to Class 2_*Quadratic Average*_. Baseline neurocognitive status again strongly segregated by longitudinal class (*χ*^2^ = 89.67, *p* < 0.0001), with each baseline classification comprising at least 60% of a respective longitudinal class.

### Baseline Predictors of Trajectory Class Membership

Table 4 presents univariable longitudinal class differences on baseline characteristics. The following variables were univariably associated with longitudinal class membership at an omnibus *p* < 0.10 and were therefore considered as covariates in multinomial regression: age, cognitive reserve [WRAT4], physiologic reserve, depressive symptoms [BDI-II], and subjective cognitive symptoms [PAOFI]. Given our primary focus on peak neurocognition, Class 1_*Stable Elite*_ was used as the reference group for the multinomial logistic regression predicting longitudinal class membership (Table 5). Sex, race/ethnicity, and BDI-II scores did not relate to longitudinal class membership at *p* < 0.10 in the multivariable analysis and were accordingly removed from the model. A one standard deviation-unit higher cognitive reserve, estimated with the WRAT4 Reading subtest (1 standard deviation = 15.4), related to a 77% decrease in odds of membership in Class 2_*Quadratic Average*_ and 88% decrease in odds of membership in Class 3_*Quadratic Low*_. Each additional self-reported cognitive symptom (PAOFI) at baseline also related to a 7% increase in odds of membership in Class 2_*Quadratic Average*_ and 14% increase in odds of membership in Class 3_*Quadratic Low*_. A year older baseline age related to a 20% increase in odds of membership in Class 3_*Quadratic Low*_ (trend-level significance) and a one standard deviation-unit increase in baseline physiologic reserve (1 standard deviation = 0.10) related to a 52% decrease in odds of membership in Class 3_*Quadratic Low*_. Age and physiologic reserve did not relate to odds of membership in Class 2_*Quadratic Average*_.

## Discussion

The present study employed GMM to identify homogenous subgroups of longitudinal peak-age neurocognition in a cohort of older PWH with up to 5 years of follow-up. Consistent with our expectation that longitudinal classes would be heterogeneous, the GMM models successfully converged upon a 3-class solution that exhibited strong separation between latent classes. Importantly, GMM identified a latent subgroup of older PWH that sustained youthful levels of global neurocognitive performance across the study period (Class 1_*Stable Elite*_). The the other two latent classes on average exhibited quadratic, although relatively modest in magnitude, slopes with intermediate (Class 2_*Quadratic Average*_) and low (Class 2_*Quadratic Low*_) levels of peak global performance across the study. Baseline SA status was predictive of higher odds of membership in Class 1_*Stable Elite*_, even when baseline performance was excluded from the GMM. Furthermore, baseline biopsychosocial indicators of resilience (i.e., cognitive reserve, physiologic reserve, better subjective functioning) also combined to predict higher odds of membership in Class 1_*Stable Elite*_. Our findings generally support SuperAging in PWH as a valid construct reflecting age-related neurocognitive resilience, however the young age range of the study sample relative to most HIV-seronegative aging cohorts and the lack of systematic decline among the lower performing longitudinal classes necessitate further validation of these findings in older-aged cohorts of PWH.

The 16.8% prevalence of Class 1_*Stable Elite*_ is similar to the 17.1% prevalence of SA identified in our prior cross-sectional evaluation [21]. Baseline neurocognitive status was predictive of longitudinal class membership, however meeting SA criteria at baseline did not guarantee membership in Class 1_*Stable Elite*_. The lack of full concordance between baseline neurocognitive status and longitudinal class membership is to be expected, as some individuals should shift across classifications due to natural intra-individual variability. However, the pattern of longitudinal data in Class 1_*Stable Elite*_ does not broadly support a regression to the mean phenomenon, whereby those who started out with the highest baselines would have exhibited the steepest declines toward the mean. Rather, most individuals in Class 1_*Stable Elite*_ were classified as SA at baseline and continued to exhibit youthful performance in the up to 5 years of follow-up data. Our processing of neurocognitive data also importantly included established test corrections for practice effects [27], which can mask neurocognitive decline when left unaccounted for.

The intra-individual fluctuations and overall quadratic patterns of change within Class 2_*Quadratic Average*_ and Class 3_*Quadratic Low*_, which were statistically significant but subtle in magnitude, are more consistent with the fluctuating neurocognitive trajectories noted in prior neuroHIV studies rather than a progressive cognitive disorder (e.g., pre-Alzheimer’s disease) [50, 51]. Although these latent classes did not demonstrate systematic neurocognitive declines, the quadratic growth patterns observed in these groups may reflect cognitive instability and possibly confer risk for future decline [52]. These quadratic trajectories may also be more consistent with a regression to the mean phenomenon, whereby early declines in performance are followed by subsequent improvements toward baseline status, which in the present study reflected average or low levels of performance that were concordant with the CN and CI classifications, respectively. Survivor bias, an issue inherent to neuroHIV and aging research [53, 54], may also partially explain this pattern under the assumption that individuals in Class 2_*Quadratic Average*_ and Class 3_*Quadratic Low*_ who experienced early neurocognitive declines dropped out of the study due to worsening disease. To help mitigate this possibility, we included auxiliary predictors of missing data, which did not significantly differ by latent trajectory groups.

Importantly, it is possible that individuals in each latent class did experience systematic neurocognitive declines as would be expected with advancing age; however, this GMM classified participants based on both slope *and* intercept (i.e., the absolute values of their baseline peak-age T scores). This likely limited the ability to detect groups solely based on trajectories regardless of baseline/absolute level of cognitive functioning. Similarly, the overall sample size of individuals with declining growth trajectories and the growth parameters of these trajectories were not sufficiently large and/or distinct to be identified as a homogenous latent subgroup by GMM. With an increase in sample size, we would also anticipate an increase in the likelihood of identifying such a “declining” subgroup. Nevertheless, the current GMM approach is clinically important to understand the trajectories of PWH at these different overall levels of cognitive functioning, particularly those with superior performance. Future research in this area may consider using neurocognitive scores that reflect only the change in neurocognitive score over time compared to one’s own baseline.

The majority of longitudinal neurocognitive studies in PWH with chronic disease have focused on identifying individuals who exhibit poor/declining trajectories, but have not been designed for detection of an elite longitudinal subgroup. Cysique and colleagues noted in a recent review that these studies focused on decline significantly vary in length and operationalization of neurocognitive change [53], yet the most consistent observation is that the majority of PWH exhibit a stable/non-progressive neurocognitive trajectory while a smaller subgroup may experience a subtle yet systematic decline [53, 55]. Only a handful of studies have explicitly focused on neurocognitive change within older groups of PWH (i.e., aged 50 or older), with support for amplified risk of neurocognitive decline compared to younger PWH and older HIV-seronegative adults [5, 56].

Even fewer studies from the broader longitudinal neuroHIV corpus have employed data-driven statistical methods with intercept and slope-based subgroup identification [57–59]. Using a mixed membership trajectory model, Molsberry and colleagues characterized trajectories of a trichotomous neurocognitive classification (i.e., normal, mild impairment, severe impairment) in the Multicenter AIDS Cohort Study and identified a 3-class solution, composed of a “normal aging” (60% of the sample; low probability of mild impairment until age 60), “premature aging” (21% of the sample; mild impairment onset between age 45–50), and “unhealthy” (19% of the sample; mild impairment in 20s and 30s) subgroups [57]. The use of a trichotomous classification is congruent with many neuroHIV studies (e.g., normal, mild HAND, severe HAND), however it does not consider variability within the “normal” range and does not utilize neuropsychological test scores that reflect youthful, rather than chronological age-based, neurocognitive abilities. In a group-based trajectory analysis of CHARTER data across the full cohort (including younger aged participants), Brouillette and colleagues modeled separate trajectories of raw test scores for each of the 15 neuropsychological tests in the CHARTER battery [58]. Roughly 16% of individuals identified as “decliners” on at least one test. However, the number of optimal class solutions ranged from 6 to 12 depending on the test and class sizes were frequently lower than the recommended 5% sample size (some as small as *n* = 3), thereby limiting the interpretability of trajectory groups. While informative, it was also noted that these data-driven studies had suboptimal considerations for practice effects [53].

Individuals in Class 1_*Stable Elite*_ were slightly but significantly younger than individuals in Class 2_*Quadratic Average*_ and Class 3_*Quadratic Low*_ in univariable analysis, however these relationships were reduced to non-significance in the multinomial logistic regression. The age of 50 has been identified as a clinically-significant cut-off for increased medical risk among PWH [60], with a recent longitudinal analysis of 1,248 PWH in the National NeuroAIDS Tissue Consortium indicating that baseline global *T*-scores were strongly predictive of mortality among PWH in their mid-50s but not among younger PWH [52]. Nevertheless, the lack of robust age-related effects on longitudinal class membership may also be related to the age range of our older CHARTER cohort, which is still relatively young compared to most HIV-seronegative aging studies. Comorbidity burden may also be a better indicator of biological age in older PWH than chronological age [61], particularly given that the positive correlation between age and physiologic reserve is attenuated among older PWH [40]. This may explain why the association between younger age and Class 1_*Stable Elite*_ was substantially weakened in the multinomial regression model that included the composite physiologic reserve index, which was not correlated with chronological age (data not shown) and was a more robust predictor of Class 1_*Stable Elite*_ (relative to Class 3_*Quadratic Low*_). Similar to our prior study of SA [21], historical HIV disease factors including AIDS diagnosis, nadir CD4, estimated years of HIV disease, and ART treatment history did not significantly differ by trajectory group, suggesting that non-HIV comorbidities may be driving the current association between physiologic reserve and longitudinal neurocognition.

Higher levels of cognitive reserve, as indexed by the WRAT4 Reading subtest, were strongly associated with higher odds of membership in Class 1_*Stable Elite*_ relative to Class 2_*Quadratic Average*_ and Class 3_*Quadratic Low*_. We observed a similar relationship in our cross-sectional study of SA [21] and this is consistent with the wide body of literature indicating a protective effect of estimated premorbid intelligence in neurocognitive aging, both in PWH and healthy older adults [34, 62, 63]. Years of education was positively correlated with the WRAT4 at *r* = 0.51, indicating roughly 25% shared variance, yet years of education did not univariably differ by trajectory group. Although years of education is also thought to contribute to cognitive reserve, measures of premorbid IQ are considered stronger estimates of educational quality than total years of education completed [63], particularly in racially diverse and marginalized older adult populations [64]. Thus, the theoretically-consistent relationship of the WRAT4 with longitudinal class membership lends further support for the inclusion of premorbid intelligence estimates above and beyond years of education in the analysis and interpretation of neuropsychological test performance.

The construct validity of Class 1_*Stable Elite*_ is also supported by its relationship with fewer self-reported cognitive symptoms at baseline. This finding coupled with our previous observation of lower total PAOFI scores in SA compared to CN and CI [21] suggests that self-reported cognitive symptoms in this population of older PWH may not only discriminate neurocognitively unimpaired PWH from those with NCI, but may also be sensitive to subclinical differences in neurocognition within the unimpaired range of performance. Self-reported cognitive symptoms are also strongly correlated with depressive symptoms, including in our sample (data not shown), yet BDI-II scores were only lower in Class 1_*Stable Elite*_ relative to Class 3_*Quadratic Low*_ in univariable but not multivariable analysis. We previously reported cross-sectional associations between SA and fewer depressive symptoms that persisted in multivariable analysis [21], however PAOFI total scores were not examined in the same multivariable model.

The present study is not without limitations. Although utilizing preexisting CHARTER data allows us to efficiently address questions pertaining to neurocognitive resilience in older PWH, we are limited by pre-defined CHARTER study parameters. Specifically, CHARTER did not enroll HIV-seronegative comparison participants, which precludes us from examining how neurocognitive trajectories differ by HIV serostatus. CHARTER did not collect data regarding certain modifiable lifestyle behaviors (e.g., diet, exercise) [65, 66] and positive psychological factors (e.g., grit, optimism) [67, 68] that could potentially inform future interventions targeting biopsychosocial resilience factors (e.g., physiologic reserve, mood) in PWH. Similarly, limited data was available regarding social determinants of health (e.g., early life adversity, housing and food security, neighborhood characteristic) that help explain racial/ethnic disparities in neuropsychological test performance. The CHARTER test battery normative procedures include race/ethnicity as a proxy for these social determinants of brain health factors, and although this aids in adjusting for premorbid influences that are independent of HIV-related CNS dysfunction, it is less desirable than directly adjusting for the social factors that are driving racial/ethnic differences in test performance. The baseline age range (50–68 years) and 5-year longitudinal timeframe, which is comparable to other longitudinal aging studies in PWH, reflects a period of enhanced vulnerability to HIV-related neurocognitive difficulties but may not capture enough individuals who have reached an age-related threshold for progressive neurocognitive decline. There are alternate methods of assessing longitudinal neurocognitive change that have been utilized in other studies among PWH, such as regression-based summary change scores, repeated-measures ANOVA, and linear mixed-effects models. While these techniques have merit, GMM is more flexible as it relates to complex non-linear trajectory modeling, robustness to violations of normality, and simultaneous estimation of latent continuous (i.e., growth factors) and latent categorical (i.e., trajectory classes) variables [69]. Latent profile analysis or cluster analysis of change scores, both within CHARTER and other cohorts, have identified domain-specific patterns of neurocognitive change across two timepoints in PWH [70, 71]. Although characterization of domain-based trajectories was beyond the scope of the present study, some neurocognitive domains are more vulnerable to aging than others (e.g., crystallized vs. fluid skills) and future work should utilize GMM to identify dissociable domain-specific trajectory patterns.

Taken together, the present study provides a novel contribution to the field of neuroHIV and neurocognitive aging research. Our results indicate that stable and youthful neurocognitive functioning is possible for older PWH, despite the inherent neurocognitive risks associated with aging with a chronic illness. HIV disease factors did not differ across longitudinal classes, whereas perceived cognitive difficulties and markers of cognitive and physiologic reserve were predictive of longitudinal class membership. These results may help elucidate the biopsychosocial mechanisms underlying neurocognitive resilience in the context of chronic HIV disease, which could help promote optimal neurocognitive aging in the rapidly growing population of older PWH.

## Figures and Tables

**Table 1:** Criteria for physiologic reserve index

Variable	Deficit criteria
Clinical measurements	
1. Abnormal BMI	> 25 or < 18 kg/m^2^
2. Low white blood cell count	< 4000 cells/μl
3. Abnormal MCHC	Male: < 27.8 or > 33.8; Female: < 26.9 or > 33.3
4. Abnormal BUN	< 8 mg/dl or > 23 mg/dl
5. Abnormal creatinine	< .6 mg/dl or > 1.2 mg/dl
6. Abnormal calcium	< 9.2 mg/dl or > 10.8 mg/dl
7. Abnormal chloride	< 96 mEq/l or > 106 mEq/l
8. Abnormal total protein (serum)	< 6 mg or > 7.8 mg
9. Low albumin (serum)	< 3.5 mg
10. Elevated fibrinogen	> 3.25
11. Low eGFR	< 60
12. Low hemoglobin	Male: < 12 μmol/l; Female: < 10 μmol/l
13. Elevated AST	> 31 U/l
14. Elevated ALT	> 31 U/l
15. Abnormal ALP	< 38 U/l or > 126 U/l
16. Abnormal potassium	< 3.5 or > 5.3 mEq/l
17. Elevated total bilirubin	> 1.1 mg/dl
18. Elevated triglycerides	≥ 150 mg/dl
19. Elevated total cholesterol	> 200 mg/dl
20. Low HDL cholesterol	Male: < 40 mg/dl; Female: < 50 mg/dl
21. Elevated glucose	> 200 mg/dl
22. Weight loss	> 10 lbs in past year
23. Low platelets	< 150 billion/l
Comorbidities	
24. HCV	Positive
25. Diabetes mellitus	Positive
26. COPD	Positive
27. Malignancy	Positive
28. Myocardial infarction	Positive
29. Renal disease	Positive
30. Hypertension	Positive or > 130 mmHg systolic or > 85 mmHg diastolic
31. Hyperlipidemia	Positive
32. Cerebrovascular accident	Positive
33. Sensory neuropathy	Positive
34. Distal neuropathic pain	Positive
35. Smoking (ever)	Positive
HIV specific	
36. Low current CD4	< 500 cells/μl
37. Nadir CD4	< 200 cells/μl
38. Detectable plasma HIV RNA	> 40 copies/ml
39. Duration of disease	> 10 years

*ALP* alkaline phosphatase, *ALT* alanine transaminase, *AST* aspartate transaminase, *BMI* body mass index, *BUN* blood urea nitrogen, *COPD* chronic obstructive pulmonary disease, *eGFR* estimated glomerular filtration rate, *HCV* hepatitis C virus, *HDL* high-density lipoprotein, *MCHC* mean corpuscular hemoglobin concentration

**Table 2:** Baseline study sample characteristics

Variable	*n* = 184
Demographics	
Number of study visits, median [IQR]	6 [4, 9]
Age (years), mean (SD)	52.9 (3.9)
Sex (male), *n* (%)	151 (82%)
Education (years), mean (SD)	13.1 (2.6)
Race/ethnicity	
Non-Hispanic Black, *n* (%)	85 (46%)
Non-Hispanic White, *n* (%)	80 (44%)
Hispanic, *n* (%)	16 (9%)
Other, *n* (%)	3 (2%)
Neurocognition	
Neurocognitive status	
SuperAger, *n* (%)	35 (19%)
Cognitively normal, *n* (%)	87 (47%)
Cognitively impaired, *n* (%)	62 (34%)
Cognitive reserve (WRAT4), mean (SD)	93.5 (15.4)
PAOFI total score, mean (SD)	2 [0, 8]
Psychiatric	
Lifetime major depressive disorder, *n* (%)	91 (49%)
Current major depressive disorder, *n* (%)	23 (12%)
BDI-II, median [IQR]	9 [3, 19]
Lifetime substance use disorder, *n* (%)	134 (73%)
Medical characteristics	
AIDS diagnosis, *n* (%)	133 (72%)
Estimated years of disease, mean (SD)	13.3 (6.2)
Nadir CD4 count, median [IQR]	109 [19, 247]
Current CD4 count, median [IQR]	475 [341, 693]
ART status	
Currently on	150 (82%)
Past use only	21 (11%)
ART naive	12 (7%)
Undetectable plasma virus, *n* (%)	101 (57%)
Physiologic reserve, mean (SD)	0.70 (0.10)

*BDI-II* Beck Depression Inventory-II, *WRAT4* Wide Range Achievement Test Reading subtest, version 4, *PAOFI* Patient’s Assessment of Own Functioning Inventory, *IADL* instrumental activities of daily living, *ART* antiretroviral therapy

**Table 3:** Growth mixture model fit statistics

	1-class	2-classes	3-classes	4-classes
Log likelihood	− 3827.206	− 3696.376	− 3380.741	− 3263.309
AIC	7680.412	7426.752	6803.481	6576.618
BIC	7722.206	7481.406	6870.995	6656.992
Sample size-adjusted BIC	7681.032	7427.563	6804.483	6577.811
Entropy	NA	0.866	0.918	0.923
LMRT	NA	0.0431	0.2444	0.4166
Class size				
Class 1	184 (100%)	104 (57%)	31 (17%)	14 (8%)
Class 2		80 (43%)	100 (54%)	85 (46%)
Class 3			53 (29%)	66 (36%)
Class 4				19 (10%)

*AIC* Akaike information criterion, *BIC* Bayesian information criterion; *LMRT* Lo–Mendell–Rubin likelihood ratio test

**Table 4:** Baseline characteristics by latent class membership

Variable	Class 1_*Stable Elite*_ (*n* = 31)	Class 2_*Quadratic Average*_ (*n* = 100)	Class 3_*Quadratic Low*_ (*n* = 53)	Test statistic *p*	Pair-wise
Demographics					
Number of study visits, median [IQR]	5 [4, 7]	6 [4, 9]	6 [4, 9]	*F* = 2.83	0.062
Age (years), mean (SD)	51.7 (3.6)	52.6 (3.4)	54.3 (4.7)	*F* = 5.37	0.005 2, 3 > 1
Sex (male), *n* (%)	27 (87%)	80 (80%)	44 (83%)	*χ*^2^ = 0.89	0.640
Education (years), mean (SD)	12.8 (2.8)	13.0 (2.6)	13.5 (2.6)	*F* = 0.94	0.391
Race/ethnicity				FET	0.975
Non-Hispanic Black, *n* (%)	14 (45%)	48 (48%)	23 (43%)		
Non-Hispanic White, *n* (%)	15 (48%)	40 (40%)	25 (47%)		
Hispanic, *n* (%)	2 (7%)	10 (10%)	4 (8%)		
Other, *n* (%)	0 (0%)	2 (2%)	1 (2%)		
Neurocognition					
Neurocognitive status				*χ*^2^ = 100.26	< 0.001
SuperAger, *n* (%)	20 (65%)	15 (15%)	0 (0%)		1 > 2 > 3
Cognitively normal, *n* (%)	11 (35%)	63 (63%)	13 (25%)		2 > 1 > 3
Cognitively impaired, *n* (%)	0 (0%)	22 (22%)	40 (75%)		3 > 2 > 1
Cognitive reserve (WRAT4), mean (SD)	98.8 (11.8)	94 (14.4)	89.2 (18.1)	*F* = 4.05	0.019 1 > 3
PAOFI total score, median [IQR]	1 [0, 6]	2 [0, 7]	2 [0, 12]	*F* = 2.94	0.056 3 > 1
Psychiatric					
Lifetime major depressive disorder, *n* (%)	16 (52%)	49 (49%)	26 (49%)	*χ*^2^ = 0.97	0.966
Current major depressive disorder, *n* (%)	3 (10%)	13 (13%)	7 (13%)	*χ*^2^ = 0.86	0.860
BDI-II, median [IQR]	8 [3, 14]	9 [3, 19]	12 [5, 26]	*F* = 2.55	0.081 3 > 1
Lifetime substance use disorder, *n* (%)	27 (87%)	71 (71%)	36 (68%)	*χ*^2^ = 4.49	0.101
Medical characteristics					
AIDS diagnosis, *n* (%)	23 (74%)	76 (76%)	34 (64%)	*χ*^2^ = 2.43	0.297
Estimated years of disease, mean (SD)	14.2 (5.9)	13.1 (6.8)	13.2 (5.2)	*F* = 0.39	0.677
Nadir CD4 count, median [IQR]	87 [19, 230]	103 [14, 246]	138 [35, 253]	*χ*^2^ = 0.87	0.648
Current CD4 count, median [IQR]	541 [341, 802]	478 [339, 710]	463 [359, 619]	*χ*^2^ = 1.15	0.564
ART status				*χ*^2^ = 6.71	0.152
Currently on	23 (74%)	87 (88%)	40 (75%)		
Past use only	6 (19%)	6 (6%)	9 (17%)		
ART naive	2 (6%)	6 (6%)	4 (8%)		
Undetectable plasma virus, *n* (%)	18 (64%)	54 (55%)	29 (57%)	*χ*^2^ = 0.76	0.684
Physiologic reserve, mean (SD)	0.72 (0.08)	0.71 (0.09)	0.67 (0.11)	*F* = 3.45	0.034 1, 2 > 3

*ART* antiretroviral therapy, *BDI-II* Beck Depression Inventory-II, *FET* Fisher’s exact test, *IADL* instrumental activities of daily living, *PAOFI* Patient’s Assessment of Own Functioning Inventory, *WRAT4* Wide Range Achievement Test Reading subtest, version 4

**Table 5:** Multinomial logistic regression predicting latent trajectory group membership

Predictor	Class 2_*Quadratic Average*_			Class 3_*Quadratic Low*_		
	OR	95% CI	*p*	OR	95% CI	*p*
Age	1.06	0.88–1.26	0.540	1.20	0.99–1.45	0.058
PAOFI	1.07	1.01–1.14	0.020	1.14	1.06–1.22	< 0.001
Physiologic reserve	0.83	0.49–1.39	0.470	0.48	0.27–0.86	0.014
Cognitive reserve	0.33	0.16–0.68	0.002	0.12	0.06–0.28	< 0.001

Provided estimates are relative to classification in Class 1_*Stable Elite*_. Estimates for physiologic reserve and cognitive reserve reflect change in odds of class membership per 1 SD-unit increase

*CI* confidence interval, *OR* odds ratio, *PAOFI* Patient’s Assessment of Own Functioning total score

## Data Availability

Anonymized, de-identified derived data values will be shared upon request from any qualified investigator (https://nntc.org/content/requests).
